# Registered Nurses’ Perspectives of the Impact of a Post-registration Education Qualification on Patient Care and Clinical Practice in Cancer Care: a Qualitative Study

**DOI:** 10.1007/s13187-022-02205-4

**Published:** 2022-08-08

**Authors:** Helen Kerr, Oonagh McSorley, Monica Donovan

**Affiliations:** School of Nursing and Midwifery, Queen’s University Belfast, Northern Ireland, UK

**Keywords:** Nursing, Cancer education, Post-registration education, Impact on patient care

## Abstract

Student evaluation of teaching is routinely completed as modules and programmes of study at higher education institutions conclude. The evaluations are often focused on the educational value and experience. For programmes with healthcare professionals as students, the impact of the learning on patient care and clinical practice is not routinely captured in these student evaluations. These insights are crucial as the definitive impact of learning for many educational programmes of study for healthcare professionals is to enhance patient outcomes. The aim of this qualitative research study was to capture the impact of a post-registration Specialist Practice in cancer pathway for registered nurses in the context of Northern Ireland following completion of the programme. Eleven participants engaged in interviews in 2021 who had completed the education programme from 2013 to 2021. Two themes inductively emerged from the data which provided insights into the specific impact of the education programme on patient care and clinical practice. Theme one identified patient outcomes improved, and was related to five sub themes; development of nurse’s clinical knowledge; enhanced awareness of the holistic impact of cancer; greater understanding of patient services available; development of clinical networks; and greater decision-making ability. Theme two related to the impact of the qualification on clinical practice through an increase in their professional credibility within the multidisciplinary team in cancer services. The debate on how to capture the impact of education on patient care and clinical practice in cancer care, should consider how to routinely capture this data.

## Introduction

From nursing’s inception as a profession approximately one century ago, there has been an ongoing appraisal of the profession in response to changes in health and society [1]. One aspect of this appraisal is the global interest in supporting nurses to extend their practice beyond the level of initial registration [2]. Advanced Practice Nursing, sometimes abbreviated to APN, is an umbrella term for nurses practicing at a higher level [3]. Advanced Practice Nursing includes advanced nursing interventions ‘that influence clinical healthcare outcomes for individuals, families and diverse populations’ [1, p.6]. The key aim of Advanced Practice Nursing is to improve patient outcomes [1]. One aspect of Advanced Practice Nursing is the availability of Advanced Practice Nurse roles. An ‘Advanced Practice Nurse is a generalist or specialised nurse who has acquired, through additional graduate education, the expert knowledge base, complex decision-making skills and clinical competencies for Advanced Practice Nursing’ [1 p. 6]. There are multiple Advanced Practice Nurse roles with one study identifying 52 different roles in 26 countries [4]. Roles include the Clinical Nurse Specialist (CNS), Nurse Consultant and Nurse Practitioner. Studies report positive patient outcomes associated with these roles in cancer services such as increased patient satisfaction, improvements in psychological support for patients, and better symptom management [5, 6].

Education has a key role in developing knowledge, skills and expertise related to Advanced Practice Nursing. There are a suite of post registration programmes available across the globe in higher education institutions (HEI) which aim to equip nurses to advance their evidence-based knowledge and skills beyond initial registration, to improve patient outcomes. In the context of the UK, the regulatory body, the Nursing and Midwifery Council (NMC), is currently reviewing the standards for post registration specifically for the NMC recordable qualification in Specialist Practice with the outcome of the review due in 2022. At the School of Nursing and Midwifery, in a HEI in Northern Ireland (NI), the Specialist Practice programme is available with seven pathways, one of which is cancer nursing, leading to a NMC Specialist Practice recordable qualification. The definition of Specialist Practice is a registered nurse who can exercise ‘higher levels of judgement, discretion and decision-making in clinical care’ relating to four broad areas; clinical practice; care and programme management; clinical practice development; and clinical practice leadership [, p. 4–5]. Nurses with the Specialist Practice qualification often progress to work in Advanced Practice Nurse roles such as a CNS. Working within a specialism such as cancer, involves nurses developing and expanding their knowledge and skills in a selected area within the discipline of nursing [7, 8]. In NI, to use the terminology ‘specialist’ in a nursing role title, a Specialist Practice qualification is recommended [9]. The Specialist Practice programme in NI is commissioned by the Department of Health in the five Health and Social Care Trusts (HSCTs) and is a 2-year part time, or 1-year full time education programme. The programme involves a 50% academic component and a 50% clinical component. The academic component involves six modules completed in the university setting and the clinical component involves each student being allocated a Practice Assessor, a registered nurse who is clinically based, and an Academic Assessor based at the university to support the student to meet the clinical competences. Another aspect of the clinical component involves clinical placements, the quantity and location of which is based on the student’s previous clinical experience. The clinical component involves the completion of a clinical portfolio to demonstrate that students have met the competences associated with the clinical component.

Student evaluation of teaching (SET) is common practice in HEI’s globally to gather information about teaching effectiveness [10, 11]. There are ongoing debates about various aspects of SETs such as student motivation to complete [10], if SET are used to improve teaching [12], and incentivising students to complete SET [11]. Evaluating the Specialist Practice in cancer pathway education programme is completed by students informally throughout, and formally at the completion of the six academic modules through an online questionnaire in the university setting, to determine the educational quality, and student experience [11]. However, the impact of this post registration education programme on patient care and clinical practice, is not routinely gathered. Standardised module evaluations are often used across HEIs [13] with recommendations for the development of national education metrics in the UK which provides educational data that is linked to patient outcomes [14]. Questions in SET tools often focus on whether educational materials supported learning, what worked well in the module and programme, and recommendations for improvements. As post registration programmes of study for healthcare professionals ultimately aim to improve patient outcomes, the primary aim of this research was to evaluate the impact of the education programme on patient care and clinical practice, after the completion of the education programme.

## Methods

### Setting

Two of the five HSCTs in NI were selected as the two settings for data collection. The rationale for this selection was that 79% of nurses commissioned by the Department of Health, NI, onto the Specialist Practice in cancer programme at a HEI in Northern Ireland from 2013 to 2018, were employed at these two HSCTs. Data collection was due to commence in May 2019 with ethical approval secured (HKerr.SREC_May19_V2), however, due to the COVID19 global pandemic, a decision was made to delay data collection. Amendments were submitted to the University Faculty Research Ethics Committee and to the two HSCTs for research governance approval to undertake data collection in 2021, and these amendments were approved.

### Study Design

A phenomenological qualitative approach was adopted as this study aimed to capture the individual’s lived experiences within their world [15]. To achieve this, questions such as ‘what is this experience like?’ and ‘what does this experience mean?’ were asked [16] in semi-structured interviews. Inclusion criteria included registered nurses who were currently working in one of two HSCTs and had completed the Specialist Practice programme, cancer pathway, leading to the NMC recordable qualification from 2013 to 2021. 9–18 participants were anticipated as the sample size in which data saturation was anticipated to be achieved.

### Sampling

Purposive sampling was adopted applying the inclusion criteria. Nurses who completed the education programme in another HEI between this timeline and currently working in one of the two HSCTs, could be recruited through snowball sampling. An email of invitation and Participant Information Sheet were forwarded to potential participants using their university student email by the lead researcher (HK), although it was recognised that previous students may no longer be accessing their university email accounts. To enhance recruitment, a gatekeeper at the two HSCTs forwarded invitations to participate and a Participant Information Sheet to registered nurses who met the inclusion criteria, using their work email address. Interested potential participants were invited to contact the lead researcher directly with questions and/or arrange a time for the interview. If there was no response within 10–14 days, a reminder email was forwarded to the nurse’s work email account and university student email account. To facilitate a cooling off period, after initial contact via email or telephone, potential participants were provided with one further week prior to the interview date.

### Data Collection and Analysis

Data collection involved online semi-structured audio-recorded interviews using the Microsoft Teams platform in July and August 2021. An online platform was selected to avoid face to face contact with frontline nurses due to the Covid-19 pandemic. Interviews were planned to last approximately 60 min. Electronic informed consent was obtained prior to the commencement of the interview after addressing any questions. Interviews were facilitated by the lead researcher (HK). To minimise any potential conflict of interest, participants were also given the option of interviews being completed by a co-researcher (OMcS), as the lead author was the Pathway Lead for the programme, for cohorts completing after 2019. Following a scoping review of the literature, the interview schedule was co-produced with the research team and a previous student. Braun and Clarkes six staged framework was used for data analysis [17]. A descriptive thematic analysis was adopted. The NVIVO computer package assisted in the organisation and coding of data. Independent blind analysis was completed among three researchers (HK, MD, OMcS) to validate interpretation in the data analysis process with consensus being reached on the themes emerging.

### Ethical Considerations

There are four main guiding ethical principles when to consider when conducting research; autonomy, beneficence, non-maleficence and justice [18]. These ethical principles were considered and implemented at all stages. Electronic written informed consent was secured prior to data collection.

## Results

Eleven registered nurses consented to participate, and data saturation was considered to have been achieved. Descriptive data on the participants is captured in Table 1. No participants were recruited through snowball sampling and all participants consented for the lead researcher (HK) to facilitate the interview. Interviews lasted an average of 45 min with a range of 31 and 71 min. Two themes inductively emerged from the data; theme one is associated with the impact of the post registration qualification on patient care, and theme two related to the impact on clinical practice.

**Table 1:** Descriptive data

Years qualified	Four to 23 years
Years working in cancer care	Four to 20 years
Current role	Clinical Nurse Specialist × 6 Oncology Nurse Practitioner × 1 Clinical educator × 1 Cancer clinical trials × 1 Acute Oncology and Haematology Service × 1 Outpatient setting × 1
Years working in current role	Four months to six years
Year completed Specialist Practice qualification	2014 × 2 2019 × 3 2020 × 5 2021 × 1

### Themes

#### Theme One: Impact on Patient Care

Theme one relates to the impact of the Specialist Practice qualification on patient care with five subthemes identified: development of clinically relevant knowledge; enhanced awareness of the holistic impact of cancer; greater understanding of patient services available; development of clinical networks; and greater decision-making ability.

##### Development of Clinically Relevant Knowledge

All participants reported a development in their clinical knowledge. This included, but was not limited to, the range and detail of cancer treatments available and symptom management. This new knowledge was reported to enhance patient care by providing a concrete evidence-base to support patients with information on the effects and potential side effects of cancer treatments, and a greater capacity to assess and manage disease and treatment related symptoms, leading to a reported increase in the nurses’ level of competence and confidence.

* ‘… it has given me a lot more knowledge and insight into even just cancer and treatments and symptoms. And it just gave me that more in depth knowledge that I wouldn’t have had, and hadn’t necessarily already got in my job. And then obviously the more I know, the more information I can give to my patient.’ (07).*

As the participant’s knowledge developed, their competence was enhanced leading to their sense of confidence growing in providing patient care. Nurses reported they were more confident in communicating with multidisciplinary team members and their sense of self-efficacy grew in advocating for patients.

*‘…If maybe medical staff have wanted to do something and I don’t really think it’s the best thing for the patient, I would certainly have the confidence … not to challenge them in a bad way, but just to say, would you think…we might be able to do it this way? Or the rationale for maybe doing it this way.’ (05).*

##### Enhanced Awareness of the Holistic Impact of Cancer

The nurse’s ability to assess and provide holistic care was enhanced as a result of the education programme. Content on the holistic impact of a cancer diagnosis and person-centred care was provided by services users in the education programme, and supplemented by academics and clinically based healthcare professionals, which enhanced the nurse’s ability to more accurately empathise with patients and appreciate the holistic and individual impact of a cancer diagnosis.

* ‘…that really helped give you insight into understanding their [patient] journey and I think then you are able to care for them better as a result.’ (05).*

*‘If you can holistically assess your patient, and I think the modules certainly help you to think more holistically, and if you are looking at your patient from more of a holistic perspective, I think the patient care is definitely enhanced’ (11).*

##### Greater Understanding of Patient Services Available

Participants shared they were more informed of the range of statutory and non-statutory services available which was shared with patients and their carers, enhancing the quality of care provided. These insights were also provided through the clinical placement component.

* ‘I’m able to navigate now, and it really informed me of the role of all the CNSs and what their job consisted of, because sometimes you get zoned in, in the role that you are doing, and you don’t understand maybe what everybody else is doing.’ (04).*

##### Development of Clinical Networks

Participants shared the education programme contributed to developing important networks which led to a more streamlined and time efficient approach when referring patients and their carers to other services. These networks were developed with peer students in clinical roles throughout NI, and through meeting other practitioners while on clinical placements.

* ‘…it allows you to meet people and to create those links and to actually know the other CNSs in other areas.’ (07).*

##### Greater Decision-making Ability

Participants reported their ability to critically think in practice was enhanced which led to a greater capacity to make evidence-based complex decisions related to patient care.

* ‘You are critically thinking about things all the time. You are applying your knowledge base from what you learned…I think the critical thinking before you do things, and thinking, why am I doing this? (11).*

#### Theme Two: Impact on Clinical Practice

##### Increased Professional Credibility

Theme two focused on the impact of the education programme on clinical practice. Participants shared the qualification increased their professional credibility within the multi-disciplinary team as it provided evidence that they had the knowledge and skills required to occupy an Advanced Practice Nurse role.

* ‘It’s …like a rite of passage…It’s like, I’ve done it. I’ve done specialist practice. I feel worthy to be here now…’ (09).*

*‘I feel confident and able to call myself a Clinical Nurse Specialist and know that, yes, that is what I am, and I have done everything that I need to do, to achieve that, and to be called that..’ (07).*

This enhanced sense of professional credibility increased their professional and personal confidence as they had demonstrated resilience to overcome the academic and time challenges to complete the education programme.

*‘[There is] value added for the organisation itself as being a nurse with a specialist practice qualification, but also the value that it had on me as a person, is me being able to grow and develop as a person, and improve my confidence and my competence and being able to be a safe and effective practitioner.’ (08).*

## Discussion

Two themes emerged from the data associated with the impact of the post-registration nurse education programme, which related to the impact on patient care and clinical practice. Theme one demonstrated that nurses reported there were improvements in patient outcomes as a result of the education programme for registered nurses working in cancer services related to five key areas, highlighting the mechanisms of how the education programme was of value in improving patient care. Education programmes in HEIs are revalidated approximately every 5 years, providing an opportunity to review and update content to ensure it aligns to current evidence-based practice, research and policy. One relevant example in this education programme is the European Oncology Nursing Society (EONS) Education Framework which also outlines that specialist cancer nursing programmes provide added value in terms of patient outcomes [19]. The revalidation process involves representation from patients, carers, students, educationalists, service providers and researchers. If the revalidation process is robust and rigorous, with effective teaching methodologies employed, then findings related to theme one are not unexpected with regards a development in the student’s knowledge, enhanced awareness of the holistic impact of cancer, and a greater understanding of patient services available, as these relate specifically to the module and programme learning outcomes for this education programme. These findings align to results from a quasi-experimental research study which reported an increase in registered nurses knowledge and perceived skills in the psychosocial care of individuals with cancer, following engagement in a cancer education programme [20]. However, in returning our attention to the findings in this research, what was interesting were reports that patient care was enhanced by the development of clinical networks with other nurses on the education programme, and through contacts initiated in clinical placements. These new insights identify how education programmes meet additional outcomes not included in programme and module aims and learning outcomes, which in this study, were attributed to improving patient outcomes. Furthermore, this study reported that nurse’s ability to think critically in the clinical setting was developed as a result of the education programme, which enhanced their decision-making ability related to improving patient care. The association between critical thinking and improved decision-making in clinical practice has been established [21]. Criticality is often associated as an academic writing skill; however, it is also a fundamental component of the advanced nurse’s role as the NMC state that specialist practice nurses should exercise higher levels of discretion, judgement and decision-making [7]. These ‘higher levels’ required for safe and effective patient care, involve critiquing skills and corroborate their importance in education programmes. This study demonstrates the transferability value of critiquing skills beyond the educational environment, into the clinical setting in improving patient outcomes. These specific insights crystallise how learning from education programmes seamlessly translates into improvements in patient care and clinical practice and should be of interest to educators developing and delivering education programmes to healthcare professionals in cancer services. The second theme related to the impact of the education programme on clinical practice with nurses reporting a greater sense of validation in the role they occupied, often Advanced Practice Nurse roles. This was borne out with reports of improved competence in their clinical role and enhanced confidence in advocating for patients. This is in keeping with results from a research study which reported a cancer education course improved nurses’ confidence in caring for patients, highlighting the far-reaching benefits of education, beyond knowledge development [20]. The impact of education programmes on registered nurses in cancer services is currently an understudied area in comparison to numerous studies published on the impact of education on undergraduate nursing students.

### Limitations and Strengths

One of the limitations of this study related to data collection being undertaken in two HSCTs in the context of NI so there should be caution in the transferability of these findings. To support transferability, demographic data has been provided. The strength of this study was the in-depth exploration of the components of educational programmes which specifically impact patient care and clinical practice in cancer services. These insights will support educationalists to develop aims, learning outcomes, and module content to best support students who are healthcare professionals, to improve patient outcomes.

To conclude, SET’s are routinely completed throughout, and at the completion of modules and education programmes at HEI’s, which should be used to not only evaluate teaching, but improve teaching [12]. For education programmes with students as healthcare professionals, the ultimate aim of these education programmes are often to improve patient outcomes. Despite this, this data is not routinely captured in HEI SETs. This seems an important omission. If this data is gathered, it will shape the content and delivery of educational programmes to ensure they are fit for purpose. The rationale for this research was to capture this data retrospectively, however, this approach would not be feasible to globally implement in HEI’s due to the time required to secure ethical and research governance approvals. Consequently, we recommend that educationalists review question items in module and programme SET to ensure there is a focus on the impact of education programmes on patient care and clinical practice, rather than the narrower focus on the educational experience. Whilst the data collected is often through a questionnaire and limited to one point in time, it will still capture useful insights into the present and anticipated outcomes in applying learning to practice, focusing on the impact on patient care and clinical practice. This data could be supplemented with discussions with students to capture the application of learning to practice. The debate on how to capture the impact of education on patient care in cancer care, should consider how to routinely capture this data, as this must be a more accurate measurement of learning.
